# A longitudinal study on perceived health in cardiovascular patients: The role of conscientiousness, subjective wellbeing and cardiac self-efficacy

**DOI:** 10.1371/journal.pone.0223862

**Published:** 2019-10-17

**Authors:** Carmen Tabernero, Tamara Gutiérrez-Domingo, Michele Vecchione, Esther Cuadrado, Rosario Castillo-Mayén, Sebastián Rubio, Alicia Arenas, Javier Delgado-Lista, Pablo Jiménez-Pérez, Bárbara Luque

**Affiliations:** 1 Instituto Maimónides de Investigación Biomédica de Córdoba (IMIBIC), Hospital Reina Sofía, University of Córdoba, Córdoba, Spain; 2 Instituto de Neurociencias de Castilla y León (INCYL), University of Salamanca, Salamanca, Spain; 3 Department of Psychology, University of Córdoba, Córdoba, Spain; 4 Department of Psychology, “Sapienza” University of Rome, Rome, Italy; 5 Department of Social and Experimental Sciences, University of Córdoba, Córdoba, Spain; 6 Department of Psychology, University of Seville, Seville, Spain; 7 Department of Medicine, University of Córdoba, Córdoba, Spain; 8 CIBER Fisiopatología de la Obesidad y Nutrición (CIBEROBN), Instituto de Salud Carlos III, Cordoba, Spain; Qazvin University of Medical Sciences, ISLAMIC REPUBLIC OF IRAN

## Abstract

Cardiovascular disease (CVD) is the world’s most prevalent chronic disease and the leading chronic cause of morbidity. There are several psychosocial factors associated with quality of life during CVD. Our main objectives were to analyze the roles of conscientiousness, subjective wellbeing and self-efficacy beliefs. The sample comprised 514 patients (mean age 63.57 years) who were assessed twice over a nine-month interval. At Time 1, participants answered a questionnaire assessing conscientiousness, perceived subjective wellbeing (positive and negative affect, life satisfaction), cardiac self-efficacy and health-related quality of life (HRQoL). The same variables (except for conscientiousness) were re-assessed at Time 2. Results showed that conscientiousness had a positive relation with subjective wellbeing, cardiac self-efficacy, and HRQoL at Time 1. Moreover, cardiac self-efficacy at Time 1 had a positive longitudinal effect on HRQoL at Time 2, while controlling for autoregressive effects. Mediation analyses indicated that the relationship between conscientiousness and HRQoL was mediated by positive affect and cardiac self-efficacy. These results suggest the usefulness of psychosocial interventions aimed at promoting positive affect and self-efficacy beliefs among CVD patients.

## Introduction

Cardiovascular disease (CVD) is the most common cause of death worldwide, although presumably it should be approximately the third most common cause of death globally [1]. It accounts for 31.5% of all deaths, and heart attacks are the main cause of death in people with CVD [2]. In Europe CVD causes about four million deaths every year, accounting for 45% of all deaths according to the European Heart Network [3]. The trend is the same in Spain, where CVD is the main cause of death, and responsible for 30% of all deaths [4].

One of the priority objectives of the Global Action Plan 2013–2020 [5] is to achieve a 25% reduction in new cases of CVD by 2025. There are several psychosocial factors in CVD that are associated with health-related quality of life (HRQoL) and according to the social cognitive theory of personality it is important to consider the interrelationships between cognitive, emotional and motivational variables [6]. The present research analyzes how a set of psychosocial determinants - conscientiousness, subjective wellbeing (affective balance; life satisfaction), and cardiac self-efficacy- affect HRQoL, testing a longitudinal model in a large sample of patients with CVD.

### Conscientiousness and health-related quality of life

As a personality trait, conscientiousness involves dutiful behavior, self-discipline, and achievement in the face of low expectations [7]. Conscientious people are healthier, as found in a study on the relationships between personality, wellbeing and health [8]. Scientific research has shown that conscientiousness is negatively associated with CVD mortality [9] and positively associated with longevity [10]. There have been numerous reports that conscientiousness is related to perceived health. For example, a review by Karademas and Tsaousis [11] concluded that people who score high for conscientiousness display a relatively large repertoire of behaviors intended to promote health (e.g., diet and exercise). All in all, studies have shown that conscientiousness predicts healthy behaviors, but the relationship is complex and there have as yet been few longitudinal studies analyzing how personality characteristics are related to cardiovascular health [8, 12]. The present study contributes to the literature by examining the association between conscientiousness and HRQoL in a large sample of cardiovascular patients. Based on the evidence reviewed above, we hypothesized that:

*H1*: *CVD patients with higher levels of* conscientiousness *will present higher HRQoL*

### Conscientiousness, subjective well-being, and health-related quality of life

Subjective well-being involves cognitive and affective evaluations about one’s life that reflect high life satisfaction, experience of pleasant emotions and low levels of negative moods. Under this definition, subjective well-being has three primary components: positive affect, negative affect and life satisfaction. These components have been consistently related to improved states of health and quality of life [13].

Earlier studies have found that conscientiousness is positively related to affective balance, and practice of health promoting behaviors [14]. There is also evidence that a positive affective balance (i.e., high levels of positive affect and low levels of negative affect), protects against CVD and is associated with the slower progression of CVD [15]. Correspondingly, it has been shown that negative affective balance is associated with a worse prognosis in cardiac patients [16]. Using an intervention program, Sanjuan et al. [17] have found that negative affect was associated with a worse development of CVD, whereas positive affect was associated with a better prognosis with respect to HRQoL. Peleg et al. [18] have observed that high values of positive affect and low values of negative affect are associated with an increase in self-assessed health in cardiac patients. In sum the empirical evidence suggests that conscientiousness is related to subjective well-being, which in turn, predicts better health outcomes. Based on earlier findings, we hypothesized that:

*H2*: *Conscientiousness will display a positive association with affective balance (i*.*e*., *a positive relation with positive affect and a negative relation with negative affect) (H2a)*, *and affective balance will mediate the relationships between conscientiousness and HRQoL (H2b)*.

A study of the relationships between personality traits and life satisfaction [19] found that conscientiousness is positively related to life satisfaction, defined as an individual’s evaluation of their life. An extensive meta-analysis that reviewed studies of associations between subjective wellbeing and cardiovascular functioning, which are related to health and cardiovascular events [20], also concluded that subjective wellbeing (operationalized as affective balance and life satisfaction), affects global health perception.

An extensive review of the relationships between conscientiousness behaviors and health [21] has suggested the need to explore mediators associated with complex mediational chains which appear along the life course. These authors [21] have confirmed that the mechanisms by which the mediational chains affect health—as the development of life satisfaction self-evaluation—have not been thoroughly studied; and therefore, life satisfaction self-evaluation could act as a mediator between the effect of conscientiousness on health-related quality of life. Based on these findings, we formulated the following hypotheses:

*H3*: *Conscientiousness will display a positive association with life satisfaction (H3a); life satisfaction will mediate the relationships between conscientiousness and health-related quality of life (H3b)*.

### Conscientiousness, cardiac self-efficacy and health-related quality of life

Some researchers [22] have found that cardiac patient adherence to a medication regime is influenced by cardiac self-efficacy beliefs. Self-efficacy levels for specific cardiovascular health-related behaviors, such as cardiovascular treatment and activity self-efficacy, are important determinants of cardiovascular health [23]. As discussed earlier, conscientiousness is also linked to health, because it facilitates adherence and self-discipline as regards medical recommendations, including medication regimes [24].

The relationship between conscientiousness and self-efficacy (i.e., the beliefs in one’s ability to undertake a certain course of action to attain a specific goal) has been analyzed in different domains, such as academic performance [25], where self-efficacy beliefs act as mediators of the personality—performance relationship. In the health domain, however, analysis of the relationship between personality, cardiovascular self-efficacy, and health had received little research attention.

Some studies have found evidence that conscientiousness is associated with diabetes self-efficacy [26]—in the sense of compliance with treatment and recommendation for diet and physical activity - with the intention of performing interventions that improve the subjective wellbeing and quality of life of these patients. There is also evidence that self-efficacy mediates the relationship between conscientiousness and HRQoL in patients with chronic disease [27]. A recent study [28] investigated emotional regulation and self-efficacy beliefs as potential mediators of the relationship between personality and quality of life, including HRQoL. The authors found that conscientiousness has direct and indirect effects on quality of life, mediated by emotion regulation and self-efficacy [28]. We therefore hypothesized that:

*H4*: *Conscientiousness will display a positive association with cardiac self-efficacy (H4a)*, *and cardiac self-efficacy will mediate the relationship between conscientiousness and health-related quality of life (H4b)*.

### The current study

**Fig 1:**
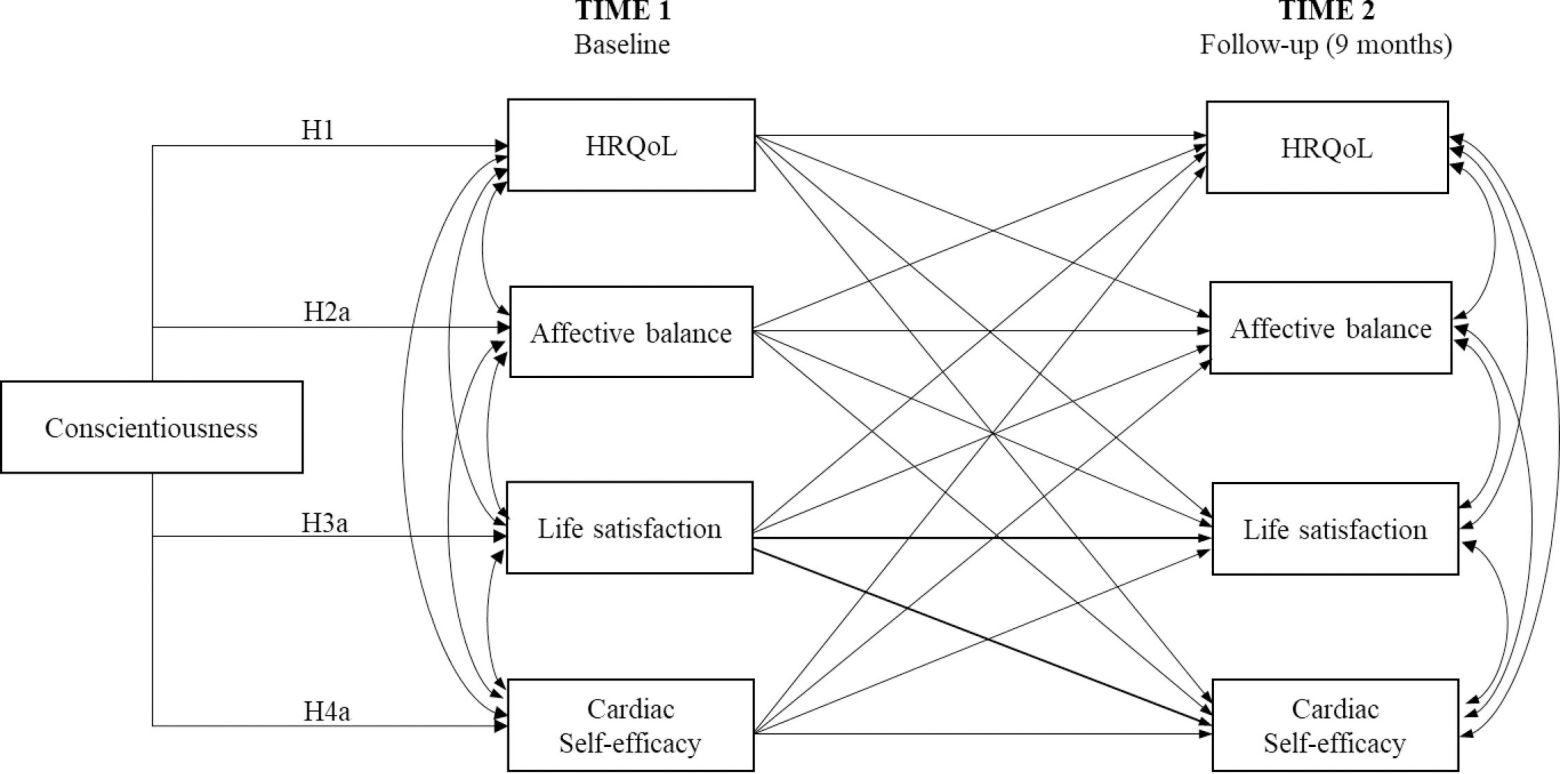
The posited model.

The main purpose of this research was to explore the pattern of relationships between conscientiousness, subjective wellbeing (affective balance and life satisfaction), cardiac self-efficacy (with respect to medicine and activity treatments) and HRQoL. The posited model is shown in Fig 1. In this model, conscientiousness affects life satisfaction, cardiac self-efficacy, positive and negative affect, and HRQoL at Time 1. It also includes autoregressive paths and cross-lagged effects for life satisfaction, cardiac self-efficacy, affective balance, and HRQoL. This allows an examination of reciprocal relations between examined variables over time.

## Method

### Participants and procedure

The current research sample comprised 514 participants (age range: 34–82 years, *M* = 63.57, *SD* = 9.16, 86.4% men). All participants were patients with CVD, who had experienced an ischemic heart disease. They were involved voluntarily in the CORDIOPREV study. CORDIOPREV’s participants were registered as being a clinical population with a high body mass index, a median of low-density lipoprotein cholesterol, and 58% presenting with a metabolic syndrome. An explanation of patient selection can be found on the project website [http://www.cordioprev.es/], indicating both the inclusion criteria (informed consent and diagnostic criteria) and the exclusion criteria (age, heart failure, ventricular dysfunction, serious risk factors, chronic diseases not related to coronary risk, participants in other studies).

The study was approved by the Research Ethics Committee of the Servicio Andaluz de Salud and the Reina Sofía Hospital (June 30^th^, 2015). Participation was totally anonymous and voluntary, and participants were informed of the objectives of the research before they provided consent for participation. Data was collected at Time 1 (since April 2016) and Time 2 (since January 2017) in a clinical room of the Hospital Reina Sofia in Córdoba (Spain). Participants used a tablet computer to respond to a series of questionnaires created using the Unipark program (v. 10.9), which is the academic program of *Questback* with online survey software. They took about an hour to complete the questionnaire in each phase (Time 1: *M* = 57'63", *SD* = 22'03"; Time 2: *M* = 64' 72", *SD* = 23' 35").

### Measures

In this section we present the scales contained in the self-reported questionnaire used in the present study. All measures except that used to assess conscientiousness were completed at both Time 1 and Time 2. The first part of the questionnaire consisted of questions collecting sociodemographic data (e.g., age, gender, educational level).

#### Conscientiousness

The conscientiousness trait was assessed using a shortened version of the *Big Five Questionnaire* (BFQ) [29]. The scale consists of 12 items, related to *scrupulousness* (e.g. ‘I've never been a perfectionist’) and *perseverance* (e.g. ‘If I fail at something, I try again until I get it’) facets. Responses to all items were given using a seven-point Likert scale, where 1 = ‘completely false’ and 7 = ‘completely true’. The 12 items were averaged to obtain an overall conscientiousness score. In the present sample, the alpha reliability coefficient was acceptable (α = .70). Because conscientiousness is a stable personality disposition, it was assessed only at Time 1.

#### Affective balance

We assessed affective balance using a short version of the *Positive and Negative Affect Scale* (PANAS) [30]. Participants responded to 10 items using a seven-point Likert scale, where 1 = ‘strongly disagree’ and 7 = ‘strongly agree’; there were five items measuring negative affect (e.g. “afraid”) and five measuring positive affect (e.g. “inspired”). In our sample, both negative and positive affect scales showed high reliability: α = .81 and α = .89, respectively at Time 1; α = .83 and α = .89, respectively at Time 2.

#### Life satisfaction

We used the *Satisfaction with Life Scale* [31] to measure the extent to which participants felt satisfied with their life and experienced subjective wellbeing. Participants responded to five items (e.g. ‘Most aspects of my life are close to my ideal’) using a seven-point Likert scale, where 1 = ‘completely false’ and 7 = ‘completely true’. The scale reliability was high (α = .86 at Time 1; α = .86 at Time 2).

#### Cardiac self-efficacy

We used a short version of the *Cardiac Self-Efficacy Scale* [32] to assess how confident participants were in their ability to follow the recommendations of their medical team. The scale consisted of 13 items divided into two factors. The first factor, *control symptoms*, composed of 8 items, and captures patient confidence in their ability to control their symptoms (e.g., ‘How confident are you that you know how to take your cardiac medication’); the second factor, *maintain function*, was composed of 5 items, and captures patient confidence that they could maintain their level of functioning (e.g., ‘How confident are you that you can maintain your usual social activities’). Responses were given using a seven-point Likert scale, where 1 = ‘not at all confident’ and 7 = ‘totally confident’. In the present study, both factors showed adequate reliability (α = .83 and α = .70 respectively at Time 1; α = .82 and α = .65 respectively at Time 2).

#### Health-related quality of life

Participant perceptions of their HRQoL were evaluated using a Spanish version of the Short-Form 12 (SF-12) health survey [33]. Participants responded to a 12-item short-form (e.g. ‘In general, how would you say your health is?’). This internationally used measure had showed good psychometric values for evaluating subjective health functions in cardiac patients [34]. Reliability was high (α = .81 at Time 1; α = .82 at Time 2).

### Statistical analysis

Descriptive statistics (means and standard deviations) were calculated for all the study variables. Pearson’s correlations were used to measure the associations between them. These analyses were carried out with SPSS (v. 22). We then used structural equation modeling (SEM) in AMOS (v. 22) with Maximum Likelihood estimator (ML) to test the model represented in Fig 1. All constructs were treated as observed variables with the exception of cardiac self-efficacy, which was included as a latent factor defined by the subscales *maintain function* and *control symptoms*. Model fit was assessed with the chi-squared statistic (χ^2^), the comparative fit index (CFI), the Tucker-Lewis index (TLI), the root mean square error of approximation (RMSEA) and standardized root mean square residual (SRMR). To evaluate model fit, we followed Hu and Bentler’s recommendations [35]. These authors see CFI and TLI values greater than .95, RMSEA values lower than .08, and SRMR values lower than .05 as indicative of adequate model fit. Finally, in order to test our hypotheses about mediation effects, we used the SPSS macro PROCESS [36]. Specifically, we estimated the indirect effects and 95% bias-corrected confidence intervals using a bootstrapping resampling procedure with 10,000 replications.

## Results

Table 1 reports descriptive statistics (means and standard deviations) and Pearson correlations for the study variables. Correlations were all in the expected direction. Conscientiousness was positively related to positive affect, life satisfaction, the two dimensions of cardiac self-efficacy (maintain function and control symptoms) and HRQoL. At both Time 1 and Time 2, positive affect and life satisfaction were positively related to cardiac self-efficacy and HRQoL. Negative affect, in contrast, was negatively related to cardiac self-efficacy and HRQoL. Both self-efficacy factors were positively associated with HRQoL.

**Table 1:** Correlations, means and standard deviations of the variables studied.

Variables	1	2	3	4	5	6	7	8	9	10	11	12	13
1 Conscientiousness	1												
*Time I- Baseline*													
Subjective Wellbeing													
2 Positive Affect	.38***	1											
3 Negative Affect	-.03	-.22***	1										
4 Life Satisfaction	.28***	.45***	-.20***	1									
5 Cardiac Self-efficacy- Control Symptoms	.34***	.33***	-.11*	.22***	1								
6 Cardiac Self-efficacy- Maintain Functions	.34***	.44***	-.18***	.33***	.49***	1							
7 Health-related Quality of Life	.19***	.48***	-.31***	.39***	.30***	.46***	1						
*Time II-Follow-up (9 months)*													
Subjective Wellbeing													
8 Positive Affect	.24***	.59***	-.17***	.40***	.24***	.34***	.40***	1					
9 Negative Affect	-.07	-.29***	.48***	-.25***	-.14**	-.16***	-.30***	-.37***	1				
10 Life Satisfaction	.16***	.40***	-15**	.58***	.23***	.20***	.29***	.44***	-.34***	1			
11 Cardiac Self-efficacy- Control Symptoms	.12**	.14**	-.08	.17***	.36***	.12**	.10*	.23***	-.19**	.26***	1		
12 Cardiac Self-efficacy–Maintain Functions	.18***	.32***	-.16***	.23***	.29***	.41***	.36***	.37***	-.26***	.29***	.46***	1	
13 Health-related Quality of Life	.18***	.44***	-.27***	.39***	.26***	.33***	.62***	.52***	-.48***	.46***	.20***	.41***	1
Mean	5.01	5.08	2.70	5.13	5.91	5.48	48.06	5.12	2.38	5.26	6.00	5.67	48.31
Sd	1.01	1.18	1.23	1.28	.88	1.07	9.38	1.16	1.22	1.30	.89	.95	10.64

Note

* *p* < .05

** *p* < .01

*** *p* < .001

**Fig 2:**
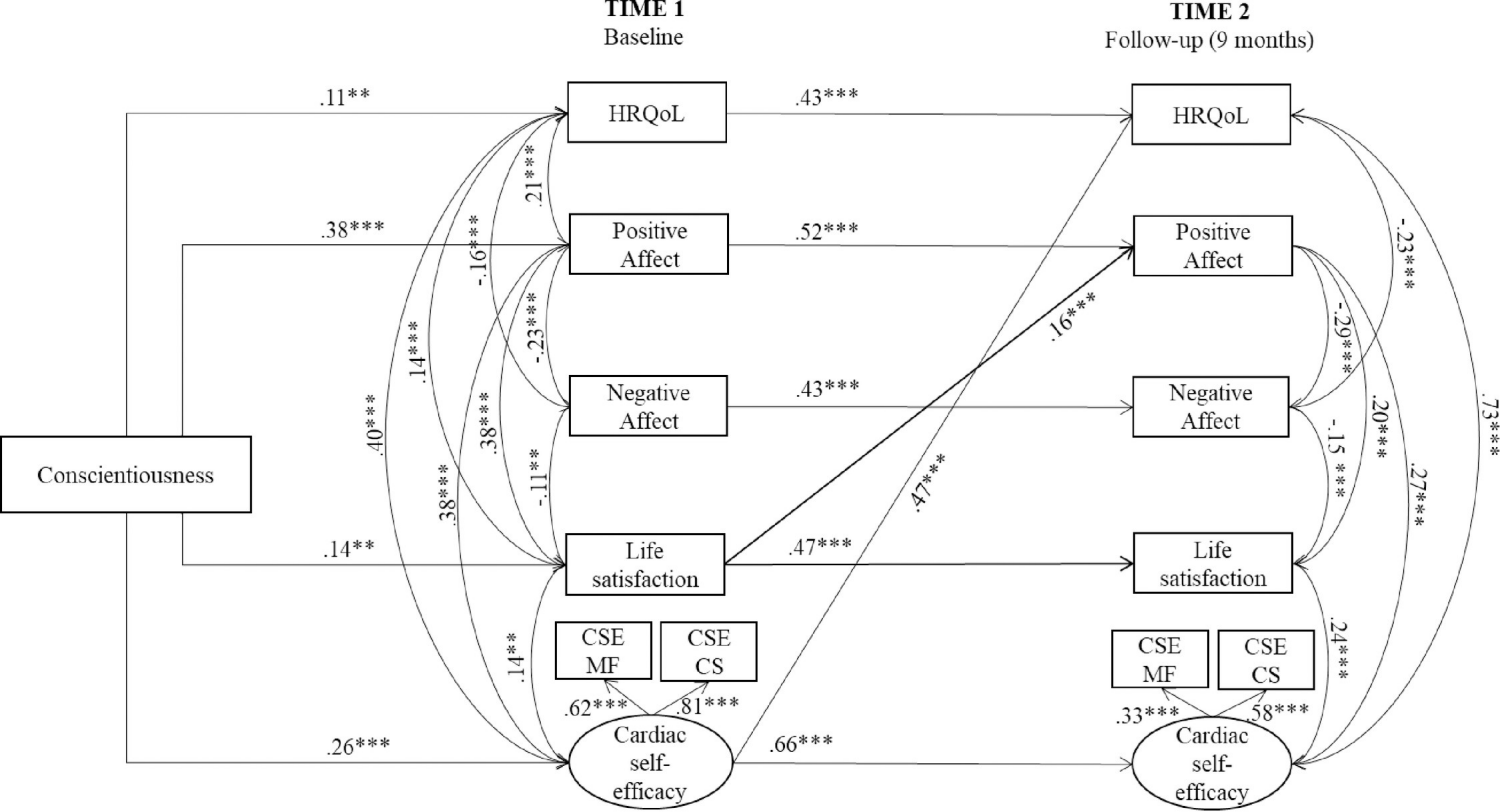
Standardized model parameter estimates (**p < .01; ***p < .001). CSE = Cardiac Self-Efficacy (MF = Maintain Functions; CS = Control Symptoms); HRQoL = Health-Related Quality of Life. For sake of clarity, non-significant paths are omitted from the figure.

The model in Fig 1 had a good fit to the data: χ^2^ (46, *N* = 514) = 145.029, *p* < .001; *CFI* = .956; *TLI* = .925; *RMSEA* = .065, 95% CI [.053, .077]; *SRMR* = .042). Standardized parameter estimates are reported in Fig 2.

As shown, conscientiousness was significantly related to HRQoL (H1), life satisfaction (H3a), and cardiac self-efficacy (H4a) at Time 1. Moreover, conscientiousness was positively related to positive affect, but showed a non-significant relationship with negative affect. Hypothesis H2a was thus only partially confirmed.

Autoregressive effects are all significant, ranging from .43 (HRQoL, negative affect) to .66 (cardiac self-efficacy). This suggests moderate to high stability over the study period for the examined variables. We found that cardiac self-efficacy at Time 1 positively predicted HRQoL at Time 2, after the stability of the variables had been taken into account. Moreover, life satisfaction positively predicted later scores on positive affect. The other path coefficients were not statistically significant.

The results of the mediation analyses (Table 2) showed that the association between Conscientiousness and HRQoL was fully mediated by affective balance (H2b). This means that those patients who have higher levels of conscientiousness feel more positive and, in turn, show higher levels of HRQoL. Satisfaction with life fully mediated the association between conscientiousness and HRQoL (H3b). Highly conscientious patients thus tend to display higher satisfaction with life, which in turn leads to higher levels of HRQoL. Lastly, the relationship between conscientiousness and HRQoL was fully mediated by cardiac self-efficacy (H4b). Conscientious patients thus had higher levels of cardiac self-efficacy scores, which in turn lead to improved HRQoL.

**Table 2:** Mediation analyses.

Coefficients for the hypotheses relating conscientiousness to affective balance (H2b)						
	Affective balance (M)			HRQoL (Y)		
	Coeff.	*SE*	*p*	Coeff.	*SE*	*p*
Conscientiousness (X)	.48	.08	< .001	.66	.43	.121
Affective balance (M)	-	-	-	2.47	.23	< .001
	*R*^2^ = .07			*R*^2^ = .21		
	*F*(1,512) = 36.17, *p* < .001			*F*(2,511) = 67.51, *p* < .001		
Indirect effect	Bootstrap (95% CI) = .164 [.114; .219]					
Coefficients for the hypotheses relating conscientiousness to life satisfaction (H3b)						
	Life satisfaction (M)			HRQoL (Y)		
	Coeff.	*SE*	*p*	Coeff.	*SE*	*p*
Conscientiousness (X)	.36	.05	< .001	.74	.44	.096
Life satisfaction (M)	-	-	-	3.10	.35	< .001
	*R*^2^ = .08			*R*^2^ = .16		
	*F*(1,512) = 43.99, *p* < .001			*F*(2,511) = 48.42, *p* < .001		
Indirect effect	Bootstrap (95% CI) = .105 [.066; .149]					
Coefficients for the hypotheses relating conscientiousness to cardiac self-efficacy (H4b)						
	Consequent					
	Cardiac self-efficacy (M)			HRQoL (Y)		
	Coeff.	*SE*	*p*	Coeff.	*SE*	*p*
Conscientiousness (X)	.31	.03	< .001	.56	.47	.241
Cardiac self-efficacy (M)	-	-	-	4.05	.58	< .001
	*R*^2^ = .15			*R*^2^ = .11		
	*F*(1,512) = 91.36, *p* < .001			*F*(2,511) = 33.18, *p* < .001		
Indirect effect	Bootstrap (95% CI) = .123 [.082; .170]					

*Note*. X = Independent variable; M = Mediator; Y = Dependent variable. Coefficients are unstandardized.

## Discussion and conclusions

The aim of this article was to explore conscientiousness, subjective wellbeing (affective balance and life satisfaction) and cardiac self-efficacy (maintain function and control symptoms) as predictors of HRQoL in a longitudinal model. We hypothesized that CVD patients with higher conscientiousness scores would report greater HRQoL, as previously stated by Friedman et al. [12]. This hypothesis was confirmed in the present study.

We also hypothesized that CVD patients with higher conscientiousness scores would report a more positive affective balance. This was partially confirmed by the results from our study, which indicated that there was a significant relationship between conscientiousness and positive affect, but not with negative affect. We also confirmed that affective balance mediated the relationship between conscientiousness and HRQoL (H2b). This finding is in line with the suggestions of Sirois and Hirsch [15].

We further hypothesized that CVD patients with higher conscientiousness scores would display greater life satisfaction. This hypothesis was confirmed, in accordance with a recent study carried out by Heidemeier and Göritz [20]. Moreover, we found that life satisfaction mediates the relationship between conscientiousness and HRQoL. This fits with Diener et al.’s [14] view that life satisfaction affects health.

The hypothesis that CVD patients with higher conscientiousness would display higher levels of self-efficacy with respect to their ability to control symptoms and to maintain functioning was confirmed. This result is consistent with previous studies of health behavior in other contexts. For example, Fisher et al. [26] demonstrated a relationship between conscientiousness and diabetes self-efficacy. We also found that the relationship between conscientiousness and HRQoL was fully mediated by cardiac self-efficacy. Importantly, beliefs in one's own capabilities have shown a longitudinal effect on HRQoL (i.e., they were able to predict later improvements in perceived health-related quality of life).

### Limitations and future research

A *limitation* of this study is the low number of women participants, which is in line with the gender imbalance in other studies [19,37]. Another limitation is that, despite the longitudinal design and very large sample, some factors that could have affected patient health between Time 1 and Time 2 were not controlled for. For instance, socioeconomic status or perceptions of social support have not been considered. Future studies should take these factors into account, as suggested by European Guidelines on CVD [38]. Conscientiousness was also assessed only at Time 1, and thus, our study provides only weak evidence for the mediation effects tested in the present sample, and the results have to be taken with caution.

One of the main *strengths of our study* is the investigation of the role played by the conscientiousness trait on CVD. This personality factor probably affects health because conscientious patients are better at following treatment recommendations. Our results might suggest the importance of providing more detailed guidelines for patients with low conscientiousness, and putting in place more comprehensive follow-up, offering more feedback. We agree with Bogg and Roberts [39] that consideration should be given to including a measure to improve conscientiousness in future programs designed to improve health, however, conscientiousness is a personality trait that could be difficult to improve with training or personalized intervention for cardiac patients. Nevertheless, based on the successful results of self-efficacy training programs on health promotion [40], training could be linked to both cardiac self-efficacy factors, such as the confidence in one’s ability to follow medical instructions and control symptoms, and to follow recommendations to maintain a level of functioning in social activities [32], for instance like that proposed in the planning program on cardiac self-efficacy used by Cajanding [41]. Another strength is the use of a longitudinal model with a large sample of CVD patients. The model includes measures of various factors that are relevant to patient health, wellbeing and quality of life. Of course, a number of other potentially relevant factors should be considered in *future research*, such as strategies for the regulation of stress. One approach to future intervention would be to study the role of such strategies in improving the HRQoL and wellbeing of cardiac patients by reducing negative affect and increasing long-term positive affect [42]. Future studies could replicate our study in other populations of chronic patients, who have to live with the symptoms in the long term, as is the case for diabetes patients. This might further increase the significance of the conscientiousness trait for patients’ quality of life. In relation to cardiovascular health, the present study supports the notion of healthy personality proposed recently by Bleidorn et al. [43].

### Psychosocial intervention

Finally, these results contribute to the identification of psychosocial variables that could be incorporated into preventive and training programs designed to reduce cardiovascular symptoms, improve overall health and prevent a second cardiac episode, all creating better HRQoL. As has been pointed out by Friedman et al. [12] it is necessary to design and analyze more sophisticated causal models that include personality traits–such as conscientiousness- and interactions amongst variables. Our results also suggest that it may be time to include wellbeing interventions in public health programs and alert policy makers to the relevance of subjective wellbeing for HRQoL. The intervention assessed by Sanjuán et al. [18] stands out as an example of an effective cardiac intervention based on positive emotions. The program developed by Schwarzer et al. [44] to improve self-efficacy with respect to dietary treatment is notable as an example of an effective self-efficacy intervention. Another example in this field is the intervention developed by Wang et al. [45]; they trained cardiac patients using a multimedia exercise program. In summary, this HRQoL model is important for future interventions with cardiac patients, as it could increase the achievement and maintenance of healthy behaviors, as suggested by the European cardiovascular prevention guide [37].

## Conclusions

The aim of this study was to develop and test a longitudinal path model linking conscientiousness, subjective wellbeing (affective balance and life satisfaction), cardiac self-efficacy and HRQoL, in patients with CVD. Our analysis of psychosocial variables allowed us to verify the existence of positive interactions and construct a predictive model for quality of life that could be used to prevent future cardiac episodes in CVD patients. Our main contribution lies in the possibility that this longitudinal model will be used to design preventive psychosocial interventions and health promotion strategies to improve the wellbeing of CVD patients. Our results indicate that it is necessary to increase the frequency with which CVD patients experience positive affect and improve their level of conscientiousness and perceived cardiac self-efficacy in order to improve their perceived HRQoL. Finally, further research is needed to confirm the associations we observed and reinforce and improve the HRQoL model.

## Supporting information

S1 Dataset The compiled data in an SPSS format (SAV). (ZIP)

## References

[1] Joseph P, Leong D, McKee M, Anand S, Schwalm JD, Tee K, et al Reducing the Global Burden of Cardiovascular Disease. Part 1: The Epidemiology and Risk Factors. Circ Res. 2017;121(6):677–94. 10.1161/CIRCRESAHA.117.308903 28860318

[2] World Health Organization Regional Office for Europe. European Health for All Database (HFA-DB). 2016. Available from: http://www.euro.who.int/en/data-and-evidence/databases/european-health-for-all-database-hfa-db

[3] Wilkins E, Wilson L, Wickramasinghe K, Bhatnagar P, Leal J, Luengo-Fernandez R, et al European Cardiovascular Disease Statistics 2017. Brussels: European Heart Network; 2017.

[4] Statistics National Institute. Deaths by death's cause. National Tables Deaths due to causes (reduced list), sex and age; 2016. Available from: http://www.ine.es/dyngs/INEbase/es/operacion.htm?c=Estadistica_C&cid=1254736176780&menu=ultiDatos&idp=1254735573175

[5] World Health Organization. Who Global NCD Action Plan 2013–2020. Geneva: WHO; 2013.

[6] Bandura A. The primacy of self-regulation in health promotion. Appl Psychol-Int Rev. 2005;54(2):245–54. 10.1111/j.1464-0597.2005.00208.x

[7] Costa PT, McCrae RR. Revised NEO Personality Inventory (NEO-PI-R) and NEO Five-Factor Inventory (NEO-FFI) manual. Odessa, FL: Psychological Assessment Resources; 1992.

[8] Friedman HS, Kern ML. Personality, Well-Being, and Health. Annu Rev Psychol. 2014;65(1):719–42. 10.1146/annurev-psych-010213-115123 24405364

[9] Jokela M, Pulkki-Raback L, Elovainio M, Kivimaeki M. Personality traits as risk factors for stroke and coronary heart disease mortality: pooled analysis of three cohort studies. J Behav Med. 2014;37(5):881–9. 10.1007/s10865-013-9548-z 24203126

[10] Burke GL, Arnold AM, Bild DE, Cushman M, Fried LP, Newman A, et al Factors associated with healthy aging: The cardiovascular health study. J Amer. Geriat Soc. 2001;49: 254–262. 10.1046/j.1532-5415.2001.4930254.x 11300235

[11] Karademas EC, Tsaousis I. The Relationship of Patient and Spouse Personality to Cardiac Patients' Health: Two Observational Studies of Mediation and Moderation. Ann Behav Med. 2014;47(1):79–91. 10.1007/s12160-013-9523-5 23780734

[12] Friedman HS, Kern ML, Hampson SE, Duckworth AL. A New Life-Span Approach to Conscientiousness and Health: Combining the Pieces of the Causal Puzzle. Dev Psychol. 2014;50(5):1377–1389. 10.1037/a0030373 23088747 PMC3651756

[13] Alessandri G, Zuffianò A, Fabes R, Vecchione M, Martin C. Linking positive affect and positive self-beliefs in daily life. J Happiness Stud. 2014;15(6):1479–1493. 10.1007/s10902-013-9487-y

[14] Sirois FM, Hirsch JK. Big Five traits, affect balance and health behaviors: A self-regulation resource perspective. Pers and Indiv Differ. 2015;87:59–64. 10.1016/j.paid.2015.07.031

[15] Boehm JK, Kubzansky LD. The heart's content: the association between positive psychological well-being and cardiovascular health. Psychol Bull. 2012;138(4):655–91. 10.1037/a0027448 22506752

[16] Meyer FA, von Kanel R, Saner H, Schmid JP, Stauber S. Positive affect moderates the effect of negative affect on cardiovascular disease-related hospitalizations and all-cause mortality after cardiac rehabilitation. Eur J Prev Cardiol. 2015;22(10):1247–53. 10.1177/2047487314549745 25208905

[17] Sanjuan P, Montalbetti T, Perez-Garcia AM, Bermudez J, Arranz H, Castro A. A Randomised Trial of a Positive Intervention to Promote Well-Being in Cardiac Patients. Appl Psychol-Hlth We. 2016;8(1):64–84. 10.1111/aphw.12062 26876425

[18] Peleg S, Drori E, Banai S, Finkelstein A, Shiloh S. The dynamic nature of self‐assessed health (SAH) as a function of negative and positive affects among cardiac patients. Appl Psychol-Hlth We. 2017;9(3):370–386. 10.1111/aphw.12099 29171195

[19] Heidemeier H, Göritz AS. The Instrumental Role of Personality Traits: Using Mixture Structural Equation Modeling to Investigate Individual Differences in the Relationships Between the Big Five Traits and Life Satisfaction. J Happiness Stud. 2016;17(6):2595–2612. 10.1007/s10902-015-9708-7

[20] Diener E, Pressman SD, Hunter J, Delgadillo-Chase D. If, Why, and When Subjective Well-Being Influences Health, and Future Needed Research. Appl Psychol-Hlth We. 2017;9(2):133–167. 10.1111/aphw.12090 28707767

[21] Shanahan MJ, Hill PL, Roberts BW, Eccles J, Friedman HS. Conscientiousness, health, and aging: the life course of personality model. Develop. Psychol. 2014;50(5):1407–1425. 10.1037/a0031130 23244406

[22] Meslot C, Gauchet A, Hagger MS, Chatzisarantis N, Lehmann A, Allenet B. A randomized controlled trial to test the effectiveness of planning strategies to improve medication adherence in patients with cardiovascular disease. Appl Psychol-Hlth We. 2017;9(1):106–129. 10.1111/aphw.12081 27779370

[23] Dorough AE, Winett RA, Anderson ES, Davy BM, Martin EC, Hedrick V. DASH to wellness: Emphasizing self-regulation through e-health in adults with prehypertension. Health Psychol. 2014;33(3):249–254. 10.1037/a0030483 23181455

[24] Molloy GJ, O'Carroll RE, Ferguson E. Conscientiousness and medication adherence: A meta-analysis. Ann Behav Med. 2014;47(1):92–101. 10.1007/s12160-013-9524-4 23783830

[25] Stajkovic AD, Bandura A, Locke EA, Lee D, Sergent K. Test of three conceptual models of influence of the big five personality traits and self-efficacy on academic performance: A meta-analytic path-analysis. Pers Indiv Differ. 2018;120:238–245. 10.1116/j.paid.2017.08.014

[26] Fisher L, Hessler D, Masharani U, Strycker L. Impact of baseline patient characteristics on interventions to reduce diabetes distress: the role of personal conscientiousness and diabetes self-efficacy. Diabetic Med. 2014;31(6):739–746. 10.1111/dme.12403 24494593 PMC4028368

[27] Axelsson M, Lotvall J, Cliffordson C, Lundgren J, Brink E. Self-efficacy and adherence as mediating factors between personality traits and health-related quality of life. Qual Life Res. 2013;22(3):567–575. 10.1007/s11136-012-0181-z 22544414

[28] Pocnet C, Dupuis M, Congard A, Jopp D. Personality and its links to quality of life: Mediating effects of emotion regulation and self-efficacy beliefs. Motiv Emotion. 2017;41(2):196–208. 10.1007/s11031-017-9603-0

[29] Caprara GV, Barbaranelli C, Bermudez J, Maslach C, Ruch W. Multivariate methods for the comparison of factor structures in cross-cultural research: An illustration with the Big Five Questionnaire. J Cross Cult Psychol. 2000;31(4):437–464. 10.1177/0022022100031004002

[30] Watson D, Clark LA, Tellegen, A. Development and validation of brief measures of positive and negative affect: The PANAS scales. J Pers Soc Psychol. 1988;54(6):1063–1070. 10.1037//0022-3514.54.6.1063 3397865

[31] Diener ED, Emmons RA, Larsen RJ, Griffin S. The satisfaction with life scale. J Pers Assess. 1985;49(1):71–5. 10.1207/s15327752jpa4901_13 16367493

[32] Sullivan MD, LaCroix AZ, Russo J, Katon WJ. Self-efficacy and self-reported functional status in coronary heart disease: A six-month prospective study. Psychosom Med. 1998;60(4):473–8. 10.1097/00006842-199807000-00014 9710293

[33] Failde I, Medina P, Ramirez C, Arana R. Construct and criterion validity of the SF-12 health questionnaire in patients with acute myocardial infarction and unstable angina. J Eval Clin Pract. 2010;16(3):569–73. 10.1111/j.1365-2753.2009.01161.x 20438603

[34] Abu HO, Ulbricht C, Ding E, Allison JJ, Salmoirago-Blotcher E, Goldberg RJ, Kiefe CI. Association of religiosity and spirituality with quality of life in patients with cardiovascular disease: a systematic review. Qual Life Res. 2018;27(11): 2777–2797. 10.1007/s11136-018-1906-4 29948601 PMC6196107

[35] Schermelleh-Engel K, Moosbrugger, Müller H. Evaluating the fit of structural equation models: Tests of significance and descriptive goodness-of-fit measures, MPR-online. 2003;8(2):23–74.

[36] Hayes AF. Introduction to mediation, moderation, and conditional process analysis: A regression-based approach 2th. ed. London: Guilford Publications; 2017.

[37] Wang LW, Ou SH, Tsai CS, Chang YC, Kao CW. Multimedia exercise training program improves distance walked, heart rate recovery, and self-efficacy in cardiac surgery patients. J Cardiovasc Nurs. 2016;31(4):343–9. 10.1097/JCN.0000000000000246 25774840

[38] Piepoli MF, Hoes AW, Agewall S, Albus C, Brotons C, Catapano AL, et al 2016 European Guidelines on cardiovascular disease prevention in clinical practice. Eur Heart J. 2016;37(29):2315–2381. 10.1093/eurheartj/ehw106 27222591 PMC4986030

[39] Bogg T, Roberts BW. Duel or diversion? Conscientiousness and executive function in the prediction of health and longevity. Ann Behav Med. 2013;45(3):400–401. 10.1007/s12160-013-9468-8 23355117

[40] Brands I, Custers M, van Heugten C. Self-efficacy and quality of life after low-intensity neuropsychological rehabilitation: a pre-post intervention study. NeuroRehabilitation. 2017;40(4):587–594. 10.3233/NRE-171446 28211828

[41] Cajanding RJ. Effects of a structured discharge planning program on perceived functional status, cardiac self-efficacy, patient satisfaction, and unexpected hospital revisits among Filipino cardiac patients: a randomized controlled study. J Cardiovasc Nurs. 2017;32(1):67–77. 10.1097/JCN.0000000000000303 26544173

[42] Gostoli S, Roncuzzi R, Urbinati S, Rafanelli C. Clinical and Subclinical Distress, Quality of Life, and Psychological Well-Being after Cardiac Rehabilitation. Appl Psychol-Hlth We. 2017;9(3):349–369. 10.1111/aphw.12098 29171196

[43] Bleidorn W, Hopwood CJ, Ackerman RA, Witt EA, Kandler C, Riemann R, Samuel DB, Donnellan MB. The healthy personality from a basic trait perspective. J Person. Social Psychol. 2019 10.1037/pspp0000231 30614724

[44] Schwarzer R, Warner LM, Fleig L, Gholami M, Serra-Majem L, Ngo J, et al Dietary planning, self-efficacy, and outcome expectancies play a role in an online intervention on fruit and vegetable consumption. Psychol Health. 2017;33(3):1–17. 10.1080/08870446.2017.1385785 28990404

[45] Gartland N, O'Connor DB, Lawton R, Ferguson E. Investigating the effects of conscientiousness on daily stress, affect and physical symptom processes: A daily diary study. Brit J Health Psych. 2014;19(2):311–328. 10.1111/bjhp.12077 24237707

