# Correction: A longitudinal study on perceived health in cardiovascular patients: The role of conscientiousness, subjective wellbeing and cardiac self-efficacy

**DOI:** 10.1371/journal.pone.0229582

**Published:** 2020-02-19

**Authors:** Carmen Tabernero, Tamara Gutiérrez-Domingo, Michele Vecchione, Esther Cuadrado, Rosario Castillo-Mayén, Sebastián Rubio, Alicia Arenas, Javier Delgado-Lista, Pablo Pérez-Martínez, Bárbara Luque

The ninth author’s name is spelled incorrectly. The correct name is: Pablo Pérez-Martínez.
